# Distress Levels among Parents of Active Duty Soldiers during Wartime

**DOI:** 10.3389/fpsyg.2017.01679

**Published:** 2017-09-26

**Authors:** Shahar Bitton, Rivka Tuval-Mashiach, Sara Freedman

**Affiliations:** ^1^Department of Psychology, Bar-Ilan University, Ramat Gan, Israel; ^2^School of Social Work, Bar-Ilan University, Ramat Gan, Israel

**Keywords:** military families, parents, Israeli army, Operation Protective Edge

## Abstract

**Objective:** Military service is a highly stressful period both for the soldiers serving and for their parents. Surprisingly, parents’ experience has been mostly ignored in the research. This study’s goal is to shed light on the experience and distress levels of parents of active duty combat soldiers during Operation Protective Edge, a military operation carried out by the Israel Defense Forces during July and August of 2014.

**Methods:** During the advanced stages of the operation, 69 parents of Israeli male combat soldiers (55 mothers and 14 fathers) completed an online survey measuring symptoms of Posttraumatic Stress Disorder (PTSD-Checklist-5) and distress (Brief Symptom Inventory-18). Participants were recruited using a convenience sample, by posting ads on the public Facebook pages of the researchers and of the groups dedicated to parents of Israeli soldiers.

**Results:** Parents’ depression and anxiety symptom levels were higher than depression and anxiety symptom levels of the adult community norms in Israel. General distress rates of parents were similar to those presented by adults in southern Israel who were exposed for 7 years to the ongoing threat of daily rocket fire from Gaza, and higher than rates of a non-threatened Israeli population. Finally, 20.2% of the parents presented PTSD-like symptoms, a higher percentage than the probable PTSD diagnosis rates that were found in the general population in Israel during previous terror waves.

**Conclusion:** This study provides preliminary evidence of soldiers’ parents’ distress and indicates the need for a better understanding of the impact of military service on soldiers’ parents.

## Introduction

Since the start of the global war on terror and increased international involvement in war zones worldwide, military service has become a common part of many young adults’ lives. For example, in 2014, out of 1.32 million active duty US army military personnel, nearly half (49.6%) were 25 years of age or younger, and 42.2% were single and had no children (U.S. Department of Defense, 2014). In Israel, where hostility with neighboring Arab countries is often manifested in periodic outbreaks of war and terror, compulsory military service is one of the most profound stages in the lives of most adolescents between the ages of 18–21 years (Mayseless, 2002). As of 2014, about 73% of male 18 years olds were recruited for a term of 3 years of compulsory military service (Pesso, 2014). Of the recruited men, about 45% served in combat units in 2013 (David, 2013).

The period of mandatory military service is considered a turbulent one, as it includes challenges and dangers that can trigger stress and distress. Soldiers deployed to war zones must make life-and-death decisions and take responsibility for the lives of others; they must also participate in life-threatening missions marked by uncertainty and danger for themselves and others (Castro and McGurk, 2007).

Military service is demanding and stressful not only for the recruited soldier, but also for his family, and specifically for his parents. Over the last 20 years, researchers have shown a great deal of interest in the experience and coping of military families after their soldier sons deploy to war zones, with a focus on soldiers’ spouses and/or children (Crow and Myers-Bowman, 2011). Family members have been found to report fear of loss and anticipatory anxiety, which can in turn lead to symptoms of distress, depression, anxiety, and reduced well-being among spouses (e.g., Rosen and Durand, 2000; Padden and Posey, 2013).

Nevertheless, despite a growing body of research on soldiers’ spouses and children, little is known about the distress and experiences of soldiers’ parents. The provision of emotional and instrumental support by parents is crucial, since in many cases soldiers are quite young, are not yet in stable romantic relationships, and have not yet formed their own families (Mayseless, 2004).

A few recent studies have focused on the experience of soldiers’ parents and have reported findings of emotional distress (Dirkzwager et al., 2005; Slaven-Lee et al., 2011) as well as feelings of fear, worry, anxiety, and helplessness during their children’s deployment (Crow and Myers-Bowman, 2011). In a qualitative study, interviews with 15 Israeli mothers regarding the upcoming recruitment of their firstborn sons raised themes of anxiety, loss of control, and feelings of ambivalence between wanting to promote independence and wanting to protect their sons (Corb, 2015, Unpublished).

The lack of research on this topic is surprising, especially given the extended literature and research findings regarding parents’ distress reactions in response to their young children’s (from childhood to adolescence) distress or trauma. The vast amount of literature in this area has focused on parents’ distress connected to the pediatric medical events of their children, including cancer (e.g., Kazak et al., 1998, 2004), burns (Hall et al., 2006), intensive care admissions (Balluffi et al., 2004; Bronner et al., 2008), organ transplantation (Young et al., 2003), asthma (Kean et al., 2006), and diabetes (Landolt et al., 2005).

The current study serves as a novel exploration of parents’ needs and distress in relation to their soldier sons during Operation Protective Edge, an Israel Defense Forces (IDF) military operation that took place in July and August of 2014.

### Operation Protective Edge

On July 8, 2014, in response to rocket attacks fired on its population centers from the Gaza Strip, the IDF launched Operation Protective Edge. Israel’s military operation began with air and artillery strikes, followed by ground forces entering Gaza (beginning on July 17). The IDF withdrew to the international border until a cease-fire agreement was implemented on August 26 (Shamir, 2015). During the operation, citizens in Israel were consistently exposed to army operations and information on casualties through intensive media coverage, both via traditional media (TV, radio) and social/online media. The fighting resulted in 74 mortalities on the Israeli side and about 2,200 mortalities on the Palestinian side (Dekel, 2014).

## Materials and Methods

### Participants

A total of 69 parents – 55 mothers and 14 fathers – of Israeli male combat soldiers participated in this study, which was conducted during the period dating from July 31 to August 25, 2014. Convenience sampling was used to recruit parents, on a voluntary basis, by posting ads on public Facebook groups initiated by parents of IDF soldiers, which invite other parents to exchange information and share their experience of having a child in the army. The selected Facebook groups were created prior to Operation Protective Edge. Ads were also posted on the public Facebook pages of the researchers. All parents who agreed to participate signed informed consent forms and completed the survey. The research was approved by the Ethics Committee of the School of Social Work at Bar-Ilan University.

### Procedure

Parents completed questionnaires online, using the QUALTRICS online survey platform^1^. A consent form, which described the research goals and requirements, was presented to participants. Only after consent was obtained the questionnaire was given to them.

### Measures

#### Psychological Distress

Brief Symptom Inventory-18 (BSI-18; Derogatis, 2001). A standardized self-report symptom inventory designed to serve as a screen for depression, somatization, and anxiety. Each statement is assessed on a 4-point Likert-type scale (0 = *not at all* to 4 = *extremely*), and responses are summed to determine a global severity index (GSI), indicating general distress. Three six-item subscales (depression, somatization, and anxiety) are averaged to indicate the score on each subscale. Derogatis (2001) reported that internal reliability for the subscales ranged from 0.74 to 0.89. In this study, Cronbach’s alphas for the BSI-18 subscales ranged from 0.73 to 0.89.

#### Posttraumatic Stress

Posttraumatic Stress Disorder (PTSD)-Checklist-5 (*PCL-5*; Weathers et al., 2013). A self-report inventory, which indicates distress levels in the past month that correspond with a DSM-V PTSD diagnosis (American Psychiatric Association, 2013). Items include symptom clusters of intrusion (five items), avoidance (two items), negative alterations in cognitions and mood (seven items), and arousal (six items). Respondents indicate how distressed they have been by each symptom over the past month by rating items on a 5-point Likert-type scale (0 = *not at all* to 4 = *extremely*). Respondents in the current study were instructed to use their sons’ IDF service as the stressful event. The original PCL has demonstrated adequate reliability (α = 0.94; test–retest *r* = 0.88) in various trauma-exposed populations, including college students (Ruggiero et al., 2003). In this study, Cronbach’s alphas for the PTSD clusters ranged from 0.85 to 0.92.

### Statistical Analysis

All statistical analyses were conducted with SPSS, Version 20. The subscales of the BSI-18 (anxiety, depression, and somatization) were compared to the adult Israeli community norms of the BSI (Gilbar and Ben-Zur, 2002), using one-sample *z*-tests. Items on these subscales are the same as those that were used in the original BSI, with the only difference being that one item was removed from the original somatization subscale and one item was removed from the depression subscale, thus making the comparison statistically relevant. The community norms are based on a nationwide representative sample of 510 community respondents (between the ages of 35 and 65 years).

The general distress rates of parents, indicated by the GSI score of the BSI-18, were compared to published GSI scores of adult residents (between the ages of 18 and 95 years) from southern Israel exposed to 7 years of daily rocket fire from Gaza (*N* = 343) and residents from a non-exposed control sample (*N* = 396) (Gelkopf et al., 2012). Inspections of skewness and kurtosis statistics revealed that GSI rates normally distributed, therefore independent *t*-tests were used for comparisons. Due to the lack of a control group in the current research, these populations from previous research studies served as relevant comparison groups and represented adult populations exposed to war-related stress and adult populations not exposed to war-related stress.

Probable PTSD of parents in the study was determined by comparing parents’ PCL-5 scores to the PCL-5 cut-off point of 33, as suggested by the National Center for PTSD (Weathers et al., 2013). In addition, in order to be eligible for a diagnosis of probable PTSD, the minimal requirement is for the individual to respond positively to one of the statements related to intrusion, one related to avoidance, two related to negative alterations in cognitions and mood, and two related to arousal (American Psychiatric Association, 2013). The threshold for a positive response (symptom endorsement) is designed to correspond to a rating of at least a moderate degree (i.e., not below the third choice out of five) (e.g., Manne et al., 1998; Brewin, 2005). Probable PTSD rates in this research were compared to probable PTSD rates of the general population in Israel during previous terror waves (Bleich et al., 2003; Gidron et al., 2004).

## Results

### Sample Characteristics

The study group consisted of 69 participants – 55 women (79.7%) and 14 men (20.3%) – with a mean age of 51.3 years (*SD* = 6.6). Comparisons of distress and PTSD-like symptoms of two genders independently weighted and unweighted did not yield a significant gender difference (*p* > 0.5).

Seven (10.1%) of the participants lived in the United States, meaning that their sons were serving as “lone soldiers” in Israel during Operation Protective Edge, while the rest lived in Israel (89.9%). Sixty-six of the participants were married (95.6%), two (2.9%) were divorced, and one (1.4%) was single. The average number of children of the parents in the sample was 4.3 (*SD* = 1.8).

### Parents’ Distress

Independent *t*-tests were used to compare the GSI scores of the current sample to GSI responses of adult residents from southern Israel who were exposed to 7 years of daily mortar fire (*N* = 343) and adult residents from a non-exposed control sample from the same geographical area (*N* = 396) (Gelkopf et al., 2012). The GSI of the current study participants (*M* = 19.7, *SD* = 12.0) was significantly higher than that of residents of southern Israel not exposed to threat (*M* = 6.4, *SD* = 9.5); *t* (463) = 10.3, *p* < 0.001. No significant difference was found between current study participants’ GSI (*M* = 19.7, *SD* = 12.0) and that of residents of southern Israel exposed to threat (*M* = 21.2, *SD* = 18.5);*t* (410) = 0.6, *p* = 0.5.

One-sample *z*-tests were used to compare the BSI-18 subscale scores to adult Israeli community norms of the original BSI (Gilbar and Ben-Zur, 2002; **Table 1**). The parents’ somatization scores did not significantly differ from community scores. Parents’ depression scores were significantly higher than known norms. Finally, parents’ anxiety scores were significantly higher than known norms.

**Table 1:** Means and SDs of BSI-18 subscales and *z*-test results in comparison to Israeli norms.

BSI-18 subscale	Current sample			Adult Israeli norms		*z*-test
	*N*	*M*	*SD*	*M*	*SD*	
Somatization	69	0.6	0.6	0.6	0.7	-0.1
Depression	69	1.1	0.8	0.7	0.7	4.8^∗^
Anxiety	69	1.6	0.9	0.8	0.7	8.9^∗^


^∗^*p < 0.001*.

Participants responses for the PCL-5 items revealed that 40 (57.9%) participants endorsed at least one intrusion symptom (Cluster B); 27 (39.1%) endorsed at least one avoidance symptom (Cluster C); 47 (68.1%) endorsed at least one symptom related to cognition or mood alterations (Cluster D); and 57 (82.6%) endorsed at least one arousal symptom (Cluster E). Given the criteria of endorsement of symptom-based clusters for a diagnosis of probable PTSD (according to DSM-V), 20 participants (28.9%) presented PTSD-like symptoms.

The PCL-5 total score presented by 21 (30.4%) participants exceeded the cut-off point suggested for probable PTSD by the National Center for PTSD (i.e., ≥33). Stricter standards, which take into consideration both the endorsement of DSM-V clusters and a total score exceeding the suggested cut-off point, showed that 14 parents (20.2%) presented PTSD-like symptoms. These PTSD prevalence rates are higher than the 10% probable PTSD rate that was found in the general population in Israel during terror waves (Bleich et al., 2003; Gidron et al., 2004). It is also worth noting that an additional 13 participants (18.8%) met the criteria for three out of the four PTSD symptom clusters.

## Discussion

This study’s main goal was to explore the experience and distress of soldiers’ parents during Operation Protective Edge, a military operation carried out by the IDF during July and August of 2014. As expected, parents of soldiers exhibited high distress and PTSD symptoms.

General distress levels, as measured by the BSI-18, were similar to distress levels of an Israeli sample exposed to the threat of ongoing rocket fire, and higher than levels of a non-threatened Israeli population (Gelkopf et al., 2012). In addition, rates of depression and anxiety symptoms were higher than for those reported in Israeli norms (Gilbar and Ben-Zur, 2002). Parents also presented PTSD-like symptoms at rates higher than those found for a probable PTSD diagnosis among the general population in Israel during terror waves in Israel (Bleich et al., 2003; Gidron et al., 2004).

As Operation Protective Edge was unexpected, the study was planned and executed at very short notice and therefore suffers from some limitations. First, the PTSD-like symptoms that were measured in this research may represent short-term distress and may not necessarily develop into PTSD. Although making a PTSD diagnosis wasn’t possible during the time of the study, the current findings indicate significant clinical distress among the parents. Previous studies have used terminology such as “probable PTSD” (e.g., Hobfoll et al., 2008) or “clinically significant PTSD-like symptoms” (Gidron et al., 2004) to refer to ongoing exposure and suggest that the reported difficulties can be understood within the broad spectrum of PTSD pathology.

In addition, while their sons were serving in the army, it might have been that parents themselves were indirectly exposed to Operation Protective Edge hostilities, through the media. There was no systematic assessment of criterion A, and it is not clear whether symptom responses were cued to a specific traumatic event. Omitting assessment of this particular criterion is common in research focused on Israeli populations (Bleich et al., 2003, 2006; Gidron et al., 2004; Hobfoll et al., 2008; Hoffman et al., 2011). Hobfoll et al. (2008, p. 13) justify this omission explicitly, saying: “We deemed it important to examine all participants as virtually everyone in Israel has at least vicarious exposure to terrorism.”

Furthermore, the current study’s sample did not include parents of children who did not take part in Operation Protective Edge and who could have served as a control group. Such a control group would have been virtually impossible to establish, given that even combat soldiers who did not enter Gaza as part of the operation were on standby to join the operation at any stage; the threat for their parents, therefore, was quite similar to that experienced by the research sample. In addition, comparing combat soldiers’ parents to parents of non-combatants is problematic because other variables – those which perhaps distinguish combat from non-combat soldiers in the first place – may be a source of different distress responses in parents. To overcome this limitation, data from previous research conducted among a large adult sample in Israel was used and served as a statistically valid comparison group.

Despite the abovementioned limitations, the current study provides significant preliminary evidence of the distress experienced by parents of IDF soldiers and specifically by parents of combat soldiers. The findings suggest that soldiers’ parents are adversely impacted by their sons’ service, especially during periods of heightened danger. More research is needed to better understand and define the specific distress response, needs, and challenges of parents. In addition, future research should examine the distress and emotional responses of mothers and fathers, so as to uncover the potential distinctions between them, in a more planned and controlled research setting. Establishing a time-frame and conducting measurements 1 month after the traumatic event can lead to a better estimation of a probable PTSD diagnosis. A potentially productive future investigation would consider the triadic interrelated relationships between the parents’ distress and emotional resources and the child’s distress and emotional resources. Adopting this kind of broader “reciprocal influences” framework could uncover the parents’ effects on the child’s distress and vice versa.

## Author Contributions

SB, RT-M, and SF contributed equally to the study conception, design, and interpretation.

## Conflict of Interest Statement

The authors declare that the research was conducted in the absence of any commercial or financial relationships that could be construed as a potential conflict of interest.

## Footnotes

1 www.qualtrics.com
